# Japanese Society for Radiation Oncology Consensus Guidelines of combined intracavitary and interstitial brachytherapy for gynecological cancers

**DOI:** 10.1093/jrr/rrac011

**Published:** 2022-03-28

**Authors:** Naoya Murakami, Tatsuya Ohno, Takafumi Toita, Ken Ando, Noriko Ii, Hiroyuki Okamoto, Toru Kojima, Kayoko Tsujino, Koji Masui, Ken Yoshida, Hitoshi Ikushima

**Affiliations:** Department of Radiation Oncology, National Cancer Center Hospital, Tokyo 104-0045, Japan; Department of Radiation Oncology, Gunma University Graduate School of Medicine, Gunma 371-8511, Japan; Gunma University Heavy Ion Medical Center, 3-39-22 Showa-machi, Maebashi 371-8511, Japan; Radiation Therapy Center, Okinawa Chubu Hospital, Okinawa 904-2293, Japan; Department of Radiation Oncology, Gunma University Graduate School of Medicine, Gunma 371-8511, Japan; Department of Radiation Oncology, Ise Red Cross Hospital, Mie 516-8512, Japan; Radiation Safety and Quality Assurance Division, National Cancer Center Hospital, Tokyo 104-0045, Japan; Department of Radiation Oncology, Saitama Cancer Center, Saitama 362-0806, Japan; Department of Radiation Oncology, Hyogo Cancer Center, Hyogo 673-8558, Japan; Department of Radiology, Kyoto Prefectural University of Medicine, Kyoto 602-8566, Japan; Department of Radiology, Kansai Medical University Medical Center, Osaka 573-1191, Japan; Institute of Biomedical Sciences, Tokushima University Graduate School, Tokushima 770-8503, Japan

**Keywords:** JASTRO Guidelines, intracavitary and interstitial brachytherapy (IC/IS), cervical cancer, brachytherapy, consensus guidelines, gynecologic cancers

## Abstract

It has been postulated that the combination of intracavitary and interstitial brachytherapy (IC/IS) is effective and safe for large and irregularly shaped uterine cervical cancer patients. However, due to its invasiveness compared to conventional intracavitary brachytherapy (ICBT), it has to be said that the implementation speed of IC/IS is slow. Until now, there have been no guidelines for required equipment, human resources, and procedural guide focusing solely on IC/IS. The purpose of this guideline is to provide radiation oncologists and medical physicists who wish to start IC/IS with practical and comprehensive guidance for a safe IC/IS introduction and to help accelerate the spread of the utilization of IC/IS nationwide. This is the English translation of the Japanese IC/IS Guidelines, and it was created in an effort to share the Japanese approach to the management of locally advanced uterine cervical cancer worldwide.

## INTRODUCTION

In the management of uterine cervical cancer with primary radiotherapy, brachytherapy plays a central role that cannot be replaced by other treatment modalities [1]. The point A prescription calculated by orthogonal 2D X-ray images based on the Manchester method became the standard method, and favorable clinical results have been reported [2]. This 2D treatment plan was summarized in detail in the ‘ Practical and QA Manual for Sealed Radioactive Source Brachytherapy Based on the Guidelines of the Brachytherapy Committee’ published by the Japanese Brachytherapy Society of the Japanese Society for Radiation Oncology (JASTRO) in 2013 [3], and has greatly contributed to the standardization and equalization of 2D-based intracavitary brachytherapy (ICBT) for cervical cancer. However, point A was not based on the individual tumor morphology. As a result, 2D methods cannot control a large or irregularly shaped tumor. Additionally, dose evaluation of organs at risk (OARs) had not been adequately performed, and only the point dose evaluation had been performed.

Since 2000, 3D image-guided brachytherapy (3D-IGBT) has been introduced in which dose calculation has been performed based on 3D images such as magnetic resonance images (MRI) or computed tomography (CT) with intra-uterine/vagina brachytherapy applicators in place [4]. In 3D-IGBT, clinical target volume (CTV) and OARs were delineated, and dose prescription and/or evaluation have been performed based on dose-volume histograms (DVHs). The 3D-IGBT has been rapidly and widely adopted also in Japan, with accumulating reports indicating that it not only enhanced local control but also decreased late toxicities when compared to the 2D era [4, 5]. The ‘Practical and QA Manual for Sealed Radioactive Source Brachytherapy Based on the Guidelines of the Brachytherapy Committee’ published in 2013 provided details on standard techniques and dose calculation for 3D-IGBT in cervical cancer [6]. In 2018, the ‘Guidelines for the Introduction of Image-Guided Brachytherapy In 2018,’ a supplement to the above-mentioned Guidelines for Sealed Radioactive Source Brachytherapy [3] was published, called ‘Guidelines for the Introduction of Image-Guided Brachytherapy (IGBT),’ which included additional information mainly on the physical and technical QA items of 3D-IGBT [7].

On the other hand, even with the use of 3D-IGBT, it has been elucidated that it is difficult to deliver an adequate tumoricidal dose while sparing surrounding OARs for bulky or irregularly shaped tumors. For such tumors, it is obvious that multi-catheter interstitial brachytherapy (MC-ISBT) should be appropriate due to the superior dose coverage and conformity compared to conventional ICBT. However, because MC-ISBT is highly invasive and requires expertise, the widespread use of this method has been limited. In such circumstances, combined intracavitary and interstitial brachytherapy (IC/IS BT) has been introduced to compensate for the disadvantage of ICBT while integrating the benefits of MC-ISBT. In IC/IS, additional interstitial needles are inserted into the area that cannot be adequately covered by the conventional ICBT, and increasing the ratio of CTV covered by the prescribed dose can be achieved while sparing OARs. IC/IS has also been gradually adopted in several Japanese hospitals, and favorable clinical outcomes have been reported not only from Western countries but also from Japanese hospitals [5, 8, 9]. This method can be adopted not only for cervical cancer but also for other gynecologic malignancies such as endometrioid carcinoma or vaginal cancer, and international recommendations advocate applying IC/IS for selective other gynecologic malignancies [10–13].

Although IC/IS is less invasive compared to MC-ISBT, several interstitial needles are used. Therefore, appropriate preparation and additional attention should be paid to avoid adverse events related to needle insertion. In addition, extra attention should be paid to dose calculations specific to IC/IS. As a result, it could be said that the speed of the spread of implementation of IC/IS is rather slow compared to the effectiveness of this method. It can easily be imagined that radiation oncologists who can perform ICBT hesitate to start IC/IS due to its invasiveness, as mentioned above. In such circumstances, there exist no guidelines for required equipment, human resources, or procedural guide focusing solely on IC/IS. It is true that there are many institutions which can perform ICBT. The purpose of this guideline is to provide radiation oncologists and medical physicists who wish to start IC/IS with practical and comprehensive guidance for a safe IC/IS introduction and help to accelerate the spread of the utilization of IC/IS nationwide.

This guideline was approved by JASTRO in July 2021. This is the English translation of the Japanese IC/IS Guidelines, and it was created in an effort to share the Japanese approach to the management of locally advanced uterine cervical cancer worldwide.

## MATERIALS AND METHODS

The members involved in the development of this guideline are radiation oncologists who specialize in gynecologic malignancies and medical physicists. A thorough literature review regarding IC/IS for gynecologic malignancies was initially performed by two radiation oncologists. Two hundred three papers were extracted by PubMed between January 2000 and December 2020 using the following keywords; ‘uterine cervical neoplasms,’ ‘endometrial neoplasms,’ ‘vaginal neoplasms,’ ‘vulval neoplasms,’ image-guided brachytherapy’ or ‘image-based brachytherapy,’ ‘intracavitary and interstitial,’ and ‘guideline.’ Among the 203 papers, 33 papers were further carefully selected, which were deemed highly important.

### Definition of IC/IS

Based on the conventional ICBT, additional interstitial needles are inserted to cover the area where adequate doses cannot be delivered only by ICBT.

### Patient selection

IC/IS should be adopted for uterine or vaginal tumors in which an adequate dose cannot be delivered to the entire high-risk CTV (CTV_HR_) and/or dose constraints for surrounding OARs cannot be observed by the conventional ICBT. In particular, CTV_HR_ > 30 cm^3^, the maximum diameter of CTV_HR_ > 4 cm, irregularly or asymmetrically shaped tumors, or thickness of vaginal wall extension >5 mm are good candidates for IC/IS [8, 14].

### Preparation

**Fig. 1.**
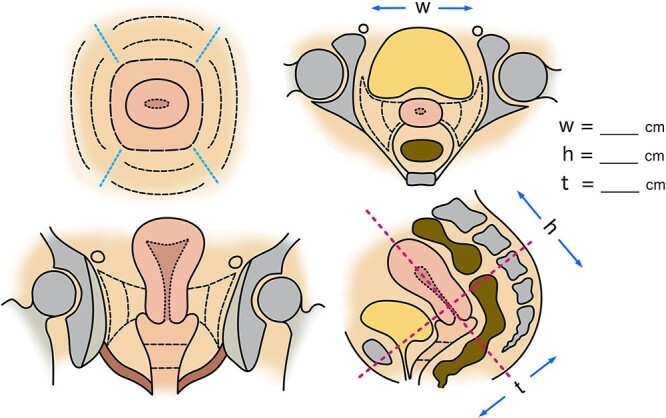
An example of a schematic template for gynecologic pelvic examination.

- Pelvic examination preferably performed under anesthesia should be done by the start of IC/IS, and it is recommended that the obtained information be drawn using a drawing template (Fig. 1).
- Pelvic MRI soon before the start of IC/IS
- Electrocardiogram
- Blood tests including blood count, biochemistry, coagulation, and cross-match for transfusion. Note the bleeding tendency in patients with platelets less than 100 000/μL. In principle, defer IC/IS in platelets less than 50 000/μL, but consider platelet transfusion if IC/IS is performed [15].
- Confirm regular medications patient takes daily that should be discontinued prior to treatment, such as antiplatelet agents and anticoagulants. Consider consulting cardiologists on the possibility of alternative medications.
- Check anesthesia history, allergy history, medical history, complications, and ease of airway clearance.
- If the intrauterine cavity is examined with a sonde at least one day before the procedure, the application on the day of the procedure will be smoother. At that time, based on the positional relationship between the intrauterine cavity sonde and the edge of the tumor using transabdominal or transrectal ultrasound (TAUS or TRUS), it is possible to predict how many additional interstitial needles will be needed in which areas to adequately cover the tumor, which can serve as a pre-plan and also help with the application on the operation day.
- Antibiotics may be considered if there is a retained pyometra and postoperative infection is highly anticipated or if a postoperative infection has occurred during previous treatment.

### Equipment required for pain control and sedation

General anesthesia or lumber spinal anesthesia managed by an anesthesiologist is preferable. Even if such anesthesia is not available, a moderate level of sedation should be performed because interstitial needle insertion is applied. It is recommended that a sedation/analgesia plan be prepared after conducting a pre-patient evaluation in advance, referring to ‘the 2020 Guidelines for Sedation and Anesthesia in Gynecologic Brachytherapy’ [16] and that the patient’s consent for sedation be obtained beforehand. The following is the equipment required for pain control and sedation.

- Sedative agents (benzodiazepines, propofol, etc.)
- Analgesics (pentazocine, opioids, etc.)
- Equipment for continuous measurement of patient vitals during the procedure
- Essential: continuous blood pressure monitor, pulse oximeter, electrocardiogram
- Recommended: Capnometer (measuring end-expiratory carbon dioxide (EtCO_2_) concentration is essential, especially in patients at high risk of upper airway obstruction, because it can detect ventilation failure at an early stage.

### Items to be used in an emergency

- Respiratory support/artificial respiration: nasal airway, Ambu bag, endotracheal tube, etc.
- Medications: Flumazenil, naloxone, catecholamine, etc., which are antagonists for an overdose of sedative/analgesic drugs.

### Equipment and applicators used

- Transrectal ultrasound (TRUS), transabdominal ultrasound (TAUS)
- X-ray fluoroscopy
- CT in the same room or in a separate room, or MRI in a separate room:

If patients need to be moved to the other room to take images, care must be taken in fixing the applicators and interstitial needles, and in the way of transferring the patient to prevent displacement of the applicators and interstitial needles. The use of a patient transfer system dedicated to brachytherapy is also beneficial.

- Applicator for intracavitary irradiation:

When performing MRI-based 3D-IGBT, use an MRI-compatible applicator; even for CT-based 3D-IGBT, an MRI-compatible applicator has fewer CT artifacts than a metal applicator. There is also an applicator for IC/IS that is equipped with an interstitial needle insertion template and with holes for interstitial needles in vaginal ovoids/ring.

- Interstitial needle:

Interstitial needles are either made of metal (reusable) or plastic (disposable). The characteristics of each material are described later.

- Examples of combined use:
  - Tandem + ovoid + interstitial needles
  - Tandem + interstitial needles
  - Tandem + vaginal cylinder + interstitial needles
  - Applicator dedicated for IC/IS, etc.

### Personnel requirements for performing IC/IS

Because IC/IS involves a more invasive technique than conventional ICBT, at least the personnel required by the guidelines for the introduction of 3D-IGBT should be secured [7]. When performing IC/IS, it is necessary to have sufficient experience with 3D-IGBT for gynecologic tumors or to perform the procedure under the supervision of a person who is proficient in 3D-IGBT.

**Fig. 2.**
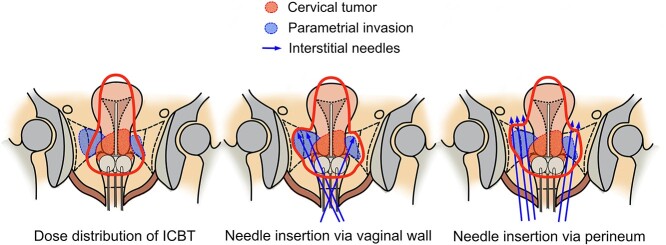
Schematic images of different interstitial needle insertion approaches.

In addition to the above, it is desirable to assign a full-time physician or nurse (person in charge of the management of sedation and analgesia) who continuously manages and monitors the patient’s condition separately from the brachytherapists.

### The medical safety system

Vaginal bleeding during and after procedures can often be stopped with tamponade with gauze. However, it is important to establish a system to obtain the cooperation of gynecologists and interventional radiologists (IVR) in advance, assuming that suturing for vaginal wall lacerations (<0.3%) [17] or angiographic arterial embolization for major bleeding during applicator removal (<2%) [18] may be required in rare cases.

### IC/IS procedure

1. Pretreatment

To prevent vomiting during sedation, abstain from food for 6 hours and water for 2 hours before the treatment. Also, explain that the patient should have defecated beforehand.

1. Sedation and analgesia

Start sedation and analgesia based on the sedation and analgesia plan prepared before treatment. Since the depth of sedation and analgesia changes over time, check the depth of sedation, analgesia, and vital signs as treatment progresses.

1. Insertion of the irradiation needle into the tissue

A) Selection of insertion route

There are two routes for insertion of the intratissue irradiation needle: the transvaginal approach and the transperineal approach (Fig. 2). The advantages and disadvantages of each are shown in Table 1. To prevent postoperative infection, thoroughly disinfect the vaginal (or perineal) site of insertion.

1. A-1) Transvaginal approach

The advantage of the transvaginal approach is that the distance until the needle reaches the tumor is shorter than that of the transperineal approach, the penetration into the tumor is much easier, and it does not require a high degree of analgesia compared to the transperineal approach. Therefore, it is a relatively easy technique to obtain. On the other hand, there is a limit to the reach of the interstitial needle due to the space limitation of the vagina, and there are cases where it is difficult to treat large tumors or tumors with complex shapes. In addition, there is interference between other vaginal applicators and the interstitial needles, which can make insertion difficult, especially in patients with narrow vaginas.

1. A-2) Perineal approach

The advantage of this approach is that it has high flexibility in terms of the reach and angulation of the interstitial needles and the extent to which they can be inserted. It can easily be used even for large tumors and tumors with complex shapes. However, because it requires a high degree of sedation, such as saddle block, and the path to the tumor is long, it should be performed under the supervision of an experienced brachytherapist at least in the beginning.

B) Selecting an interstitial needle

There are two types of materials for interstitial needles: plastic and metal. Each has advantages and disadvantages as shown in Table 2, and should be selected according to the preference of each brachytherapist and their level of proficiency in the method. Regardless of which type of needle is used, visually confirm that there is no damage to the interstitial needle before insertion, and use an appropriate obturator to maintain needle strength during insertion.

1. B-1) Plastic needle

**Table 1:** Comparison of different interstitial needle insertion approaches

	Advantages	Disadvantages
The transvaginal approach	・Needle insertion is easy because the needle path is short. ・Deep sedation is not always required.	・Difficult to adopt for patients with a narrow vagina. ・Limitation for the reach of the needles due to limited angulation. ・It is challenging to handle bulky or complicated shaped tumors.
The transperineal approach	・Wider range of the needle insertion is possible due to its flexible angulation. ・It is easier to approach bulky or complicated shaped tumors.	・Requires expertise and experience because needle path is long. ・Risk of vaginal laceration. ・Deeper sedation is needed.

**Table 2:** Comparison of different interstitial needle materials

	Advantages	Disadvantages
Plastic needle	・MRI compatible. ・Creates fewer artifacts on CT. ・Offset is shorter than metallic needle.	・Less sharp compared to metallic needles and easily be bent. ・Disposable ・Less visible on ultrasound images. ・The public insurance does not cover the needle cost.
Metallic needle	・Sharper and more robust than the plastic needle. ・Reusable ・More visible on ultrasound images.	・Cannot take MRI with metallic applicators in place. ・Creates more artifacts on CT. ・Offset is longer than plastic needle.

There are two types of plastic needles (sharp and round). Because it has only been short period of time since round plastic needles were available in Japan and not so much information or experience has accumulated regarding the round plastic needle, only sharp plastic needles are described in this guideline. When MRI-based 3D-IGBT is performed, metallic needles cannot be used unless they are made of titanium. Plastic needle has the advantage of being less artifactual, even when CT-based. They are disposable, but at the time of the development of this guideline, Japanese public insurance does not cover plastic needle costs. To determine the first stopping point in the treatment planning system, the offset value (distance from the applicator tip to the closest stopping point) obtained by the dummy source or the value obtained from commissioning performed in advance is used.

1. B-2) Metallic needle

Although the metallic needle has the advantage of easy puncture and better real-time ultrasound visualization during needle insertion, it has a sharper tip, so be careful of the risk of damaging blood vessels or bowels. In addition, the offset value is longer than that of the plastic needle, so care must be taken with the depth of the needle. To determine the first stopping point in the treatment planning system, use the offset value obtained from the dummy source or commissioning performed in advance.

C) Interstitial needle insertion procedure

The interstitial needle is inserted aiming at the tumor portion where the dose is insufficient in the dose distribution of normal ICBT. For insertion, it is essential to have an image in mind of the insertion site, angle, depth, etc., in advance using MRI images before IC/IS and TRUS or TAUS while inserting a sonde into the uterine cavity, as described before. In addition, pelvic examination findings on the day of the procedure and modalities to assist the insertion as described below are used as appropriate. The insertion is performed with the utmost care. The following is an overview of the insertion method by different approaches.

1. C-1) Transvaginal approach

First, the regular ICBT applicator insertion is performed, followed by the insertion of the interstitial needles. Although needle insertion after insertion of the ovoid is easy and the ovoid serves as a guide for the insertion site, it has the disadvantage of limiting the space and range of needle insertion due to interference with the ovoid. If the interstitial needle is inserted into the tissue before inserting the ovoid, there is more freedom in the needle insertion site and direction, but the needle must be inserted from the side of the vagina to avoid interference so that the ovoid can be inserted after insertion.

There are two methods of needle insertion from the vaginal wall: freehand insertion and insertion using the applicator for IC/IS as a template. Freehand needle insertion offers a wide range of applicability with a high degree of freedom in the insertion site and angulation, but it is difficult to ensure reproducibility. When a template (ring or ovoid with holes) is used, the insertion site and direction are restricted according to the template, but it has the advantage of high reproducibility.

When puncturing the vaginal wall, it is recommended that the vaginal wall be sufficiently stretched after the insertion of the ICBT applicator and that the needle be inserted into the vaginal wall as vertically as possible in order to avoid vaginal wall lacerations caused by the interstitial needle penetrating the vaginal wall at an angle.

1. C-2) Transperineal approach

The patient should be in the extended lithotomy position to avoid pubic bone interference. Because the ovoid and the interstitial needle may interfere with each other in the transperineal approach, it is often possible to perform the procedure smoothly by inserting the interstitial needles first and then the ICBT applicators. In some cases, the interstitial needle may be excessively bent by the ovoid inserted after the interstitial needles. In such a case, confirm that the source path is not blocked by using a dummy source and consider replacing it with a smaller size ovoid. Meanwhile, inserting the tandem first may make it easier to insert the interstitial needle because it serves as an indicator of the insertion site of the interstitial needle.

In the transperineal approach, there is a risk that the needle tip may point in an unexpected direction due to the high degree of flexibility in the angle of the interstitial needle in the tissues, so it is recommended that the needle tip be confirmed in real-time by TRUS during insertion. If TRUS is not used, it is safer to use a perineal template where the needle angle is prefixed.

To avoid damage to the vaginal wall due to the long penetration route, be careful not to expose the interstitial needle in the vagina. If the position of the needle tip cannot be confirmed by TRUS, it is safer to insert a finger into the vagina and use the finger to palpate and guide the needle tip under the vaginal mucosa as it moves deeper. If it is necessary to insert the needle into a deeper plane against the vaginal wall surface, the needle under the vaginal mucosa serves as a marker, making it easier to insert the needle into the deeper planes.

When the ICBT applicator is inserted after insertion of the interstitial needles, the interstitial needles may come out slightly. Check and modify the tip of the interstitial needle again with TRUS before vaginal gauze packing is performed.

1. C-3) Modalities that can assist interstitial needle insertion

When freehand needle insertion is performed, it is possible that the needle will go in an unintended direction. Since organ damage associated with the insertion of an interstitial needle can lead to serious adverse events such as bleeding, infection, and bowel perforation, it is necessary to perform the insertion under real-time image guidance such as TRUS with X-ray assistance or frequent use of CT to avoid unnecessary normal organ injury and to always pay attention to the position of the needle tip while inserting the needle and keeping it inside the CTV_HR_.

TAUS guidance: Inject about 100 ml of saline into the bladder to avoid the artifact of intestinal gas. Make sure that the tandem is inserted into the uterine cavity. Insert the needle while checking the needle tip. If the needle tip cannot be tracked, CT guidance should be used.

TRUS guidance: Real-time confirmation of needle position and depth is safer and has the advantage of completing the procedure more quickly than CT guidance, but unlike prostate brachytherapy under TRUS guidance, experience is required to recognize the TRUS images in the female pelvis. The range of images is limited to the depth of the transrectal probe, so the needle tip needs to be confirmed by CT or TAUS for deeper needle insertion.

CT-guided (+ X-ray fluoroscopic guidance): Since the needle insertion itself will be blind, it is necessary to advance the needle gradually and take the CT multiple times. After the position and angle of the needle are determined, the depth can be determined by real-time X-ray fluoroscopic guidance, which reduces the number of CT exposure.

1. C-4) Precautions after needle insertion

When multiple interstitial needles are used, it is possible to mistake the channel number of the transfer tube to be connected or to misidentify the channel number when the applicator is reconfigured in the treatment planning system. When multiple interstitial needles cross each other or the needle passes the air cavity of the vagina, and it is difficult to identify them, insert a dummy source that helps indicates the source path and confirm it. In addition, it is desirable to take measures such as attaching number stickers to all interstitial needles so that they can be easily identified.

If it takes a long time until the start of irradiation after the insertion is completed, consider inserting an obturator into the interstitial needle to prevent blood or disinfectant contamination. If the connection point of the interstitial needle is faulty, or if blood or other substances have contaminated the inside of the catheter and cannot be removed, cut the faulty point of the needle using a dedicated instrument. In such a case, measure the length of the interstitial needle again and reflect it in the applicator reconstruction of the treatment plan.

Needle retraction or displacement can happen, especially when patients need to be moved to another room for image acquisition. To avoid such circumstances, when needed, needle fixation using a button sutured to the skin could be considered.

D) Treatment plan image acquisition

After implantation and insertion of the ICBT applicator and the interstitial needles are completed, CT and/or MRI for treatment planning should be taken. When image acquisition is required in a room separate from the brachytherapy room, care should be taken to ensure that the positional relationship between the applicators and the interstitial needle is not displaced or retracted during transportation, such as by fixing the applicators with tape, etc. During MRI imaging, care should be taken to ensure that the interstitial needle does not move due to the weight or pressure of the MRI coil. After confirming the obtained images and confirming that the applicators and interstitial needles are in the appropriate locations and depths and that there are no excessively bent parts in the interstitial needles, move on to treatment planning.

E) Treatment Planning/Dose calculation

Dose calculation of IC/IS is performed based on the Practical and QA Manual for Sealed Radioactive Source Brachytherapy Based on the Guidelines of the Brachytherapy Committee [6] or Guidelines for the Introduction of Image-Guided Brachytherapy in 2018 [7]. In particular, in the case of IC/IS, the length to the first stopping point and the offset value differ depending on the type of interstitial needle. Therefore, attention should be paid to using the correct offset value, which is acquired with commissioning in advance.

For contouring of high-risk CTV (CTV_HR_), the GYN GEC-ESTRO (Gynaecological Groupe Europeen de Curietherapie-European Society for Radiotherapy & Oncology) contouring guidelines [19] are used for MRI-based contouring, and for CT-based contouring, the guidelines by JROSG (Japanese Radiation Oncology Study Group) [20, 21] should be referred to. The risk organs are the rectum, sigmoid colon, bladder, and, if necessary, the small intestine near the vagina and uterus.

Calculate and record the bilateral point A doses, D_98%_, D_90%_, D_50%_ for CTV_HR_ (the minimum dose covering X% of the CTV_HR_), D_0.1cm3_, D_2cm3_ for rectum, sigmoid colon, and bladder (most exposed Y cm^3^ of tissue), respectively [19]. The small intestine in close proximity to the vagina or uterus should be calculated if considered necessary.

**Fig. 3.**
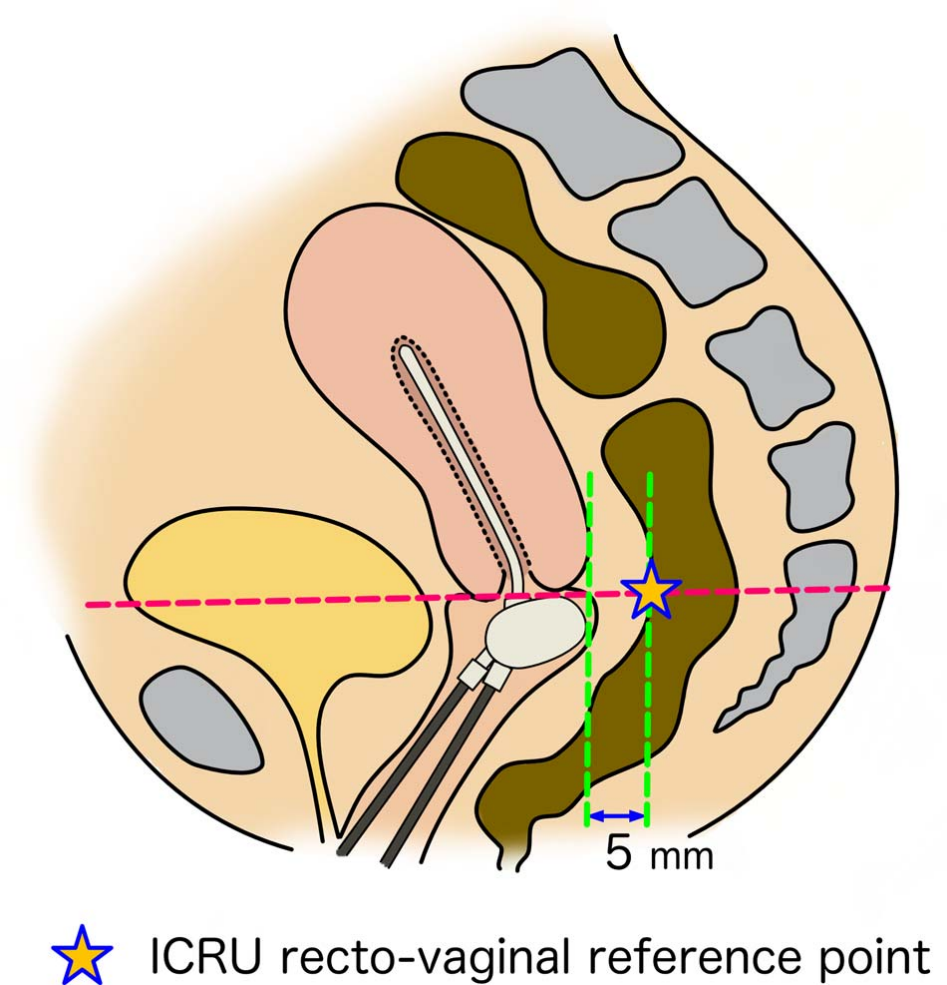
A schematic image showing International Commission on Radiation Units and Measurements (ICRU) recto-vaginal reference point. It is located 5 mm dorsal of the posterior wall on the axis perpendicular to the body axis, at the level where tandem and the vaginal source cross.

Evaluate the International Commission on Radiation Units and Measurements (ICRU) recto-vaginal reference point dose [22] (Figure 3). Vaginal D_2cm3_ has been reported to correlate with late effects and should be evaluated as needed [23, 24].

In calculating dose distributions, the standard Manchester method dose distribution based on the point A prescription is first prepared. Then, the dose distribution is modified by adding doses to the areas in the CTV_HR_ where the doses are insufficient using the interstitial needles inserted into the tissue. Such a procedure will prevent dose contribution by the interstitial needles from being extremely large. It is recommended that the total dwell time from the interstitial needles should be limited to 10–20% of the total dwell time calculated from the standard Manchester method [25–27]. Ensure that the source is not unnecessarily dwelled outside the CTV_HR_ by checking dose distribution on CT slice by slice to protect OARs.

The biological equivalent dose is calculated using the LQ model to add the dose from each IC/IS session (D90% of CTV_HR_) and the dose from whole pelvic external beam radiation therapy. The doses for the risk organs are calculated in the same way. In the GYN GEC-ESTRO guideline, α/β = 10 Gy for tumors and α/β = 3 Gy for late reactions in normal tissues are calculated in the form of the equivalent dose in 2 Gy fractions (EQD_2_). It has been reported that the sum of these doses correlates with local control. The GEC-ESTRO and American Brachytherapy Society (ABS) guidelines recommend a target CTV_HR_ D_90%_ and provide a dose constraint of D_2cm3_ rectum and bladder [28, 29].

In the standard external radiation schedule in Japan, central shielding (CS) is used as the tumor shrinks. According to the definition of D_90%_, the dose from CS is usually not added to the calculation of the total dose of EBRT and brachytherapy. However, it was postulated that the dose contribution to the tumor from CS is not negligible [30]. Against this background, it should be noted that the recommended doses of GYN GEC-ESTRO and ABS cannot be applied as they are. In Japan, the Fourth Edition of General rules for clinical and pathological study of uterine cervical cancer in Japan [31] stipulates that when CS is used, the respective doses should be recorded in parallel rather than combined. On the other hand, the combined dose of total EBRT and brachytherapy, excluding CS, has been used to guide actual daily practice as mentioned above. In a recent multicenter prospective clinical study of CT-based 3D-IGBT in Japan employing dose calculation excluding dose contribution from CS, the median combined dose for CTV_HR_ D_90%_ was 70 Gy (EQD_2_), which can be considered as an index [32].

If sedation and analgesia are not sufficiently obtained, the position of the applicator and the interstitial needle may shift due to the patient’s unintentional body movement. Therefore, monitor the patient carefully during treatment planning, and connect the applicator to the transfer tube immediately before treatment.

F) Irradiation

When connecting the interstitial needle to the transfer tube, be careful because the weight of the transfer tube can pull the interstitial needle toward the outside of the body. It would be helpful to use some kind of support stand to prevent needle displacement due to the transfer tube weight.

When multiple interstitial needles are inserted, be careful not to connect the wrong transfer tube to the wrong interstitial needle. Use a dummy source to confirm the applicator passage in advance to avoid errors during irradiation.

Visually confirm that there is no significant change in the applicator position before and after treatment.

G) Needle removal

Bleeding often occurs during needle removal, so paramount attention should be paid to bleeding during the removal of the interstitial needle.

In general, the transvaginal approach is more likely to cause bleeding than the transperineal approach because of the abundant blood flow in the vaginal wall. However, if the point of bleeding is well identified and pressure is applied to the bleeding point for a sufficient time, hemostasis can often be achieved. In addition to focused hemostasis, filling the vagina with gauze (or iodoform gauze) and applying continuous pressure to the entire vagina is also effective. If the outside of the gauze does not turn red after a while, it is likely that hemostasis has been obtained.

Even in cases where minor bleeding persists, and multiple gauze changes are required, overnight tamponade with gauze will almost always stop bleeding the next day. However, if arterial bleeding or bleeding of more than moderate severity is suspected, do not attempt to handle the situation alone, but do not hesitate to ask for help from a gynecologist or IVR physician.

Note that intra-abdominal hemorrhage may occur in rare cases when the tip of the needle is beyond the CTV_HR_ and enters the peritoneal cavity. Check vital signs and consider necessary clinical tests, including imaging studies. If intra-abdominal hemorrhage is confirmed, try to maintain blood pressure, prepare for a blood transfusion, and contact the gynecologist or surgeon for immediate help.

Confirming the position of uterine arteries by prior MRI is also useful in avoiding bleeding. Intraoperative Doppler ultrasonography is also helpful in identifying vessels in a real-time manner.

The above description focuses on IC/IS for curative cases of uterine cervical cancer. However, it is possible to apply the same procedure for other gynecological malignancies, such as postoperative vaginal recurrences thicker than 5 mm and difficult to treat with ICBT alone, uterine endometrial cancer, vaginal cancer, or vulvar cancer that has massive vaginal involvement. It is recommended that such miscellaneous gynecological tumors also be treated using the same concept and method as IC/IS [10–13].

### Effectiveness and safety of IC/IS

In 3D-IGBT for cervical cancer, randomized controlled trials comparing ICBT versus IC/IS have not yet been reported.

A large retrospective multicenter study comparing the results of both treatment methods has been conducted by the External beam radiochemotherapy and MRI-based adaptive brachytherapy in locally advanced cervical cancer (EMBRACE) group [8]. In this study, the local control rate, late adverse events, and DVH were evaluated in 300 patients in the IC/IS group and 310 patients in the ICBT group. The 5-year local control rate was 91% in the IC/IS group and 89% in the ICBT group (*P* = 0.06). The local control rate for large tumors ≥30 cm^3^ was significantly better in the IC/IS group. On the other hand, for small tumors of <30 cm^3^, there was no significant difference in local control between the two groups. There was no significant difference in late adverse events in the bladder or gastrointestinal tract between the two groups. In comparing DVH parameters, although the CTV_HR_ volume was significantly higher in the IC/IS group, the CTV_HR_ D_90%_ dose was significantly higher in the IC/IS group than in the ICBT group. Meanwhile, there was no significant difference in the D_2 cm3_ doses of the bladder, rectum, and sigmoid colon between the two groups. Therefore, IC/IS can increase the target dose without increasing the OAR dose, which was thought to lead to an excellent local control rate, especially for large tumors, without increasing adverse events.

In the EMBRACE study mentioned above, DVH parameters were compared between the tandem plus ovoid group and the tandem plus ring applicator group, and in both groups, CTV_HR_ D_90%_ dose was increased and D_2 cm3_ doses in the bladder, rectum, and sigmoid colon were decreased, respectively, with the combination of interstitial needles compared with ICBT alone [33].

A case with severe bleeding treated by transcatheter arterial embolization was reported in which an obturator artery was injured by an interstitial needle inserted using an ovoid with holes as a template. The lessons learned from this case include inserting the needle under image guidance and, if CT preplanning is used to determine needle position beforehand, paying attention to anatomical changes during the treatment period, such as tumor shrinkage due to treatment [18].

The initial treatment results of a novel applicator dedicated to IC/IS were reported from the United States. The median CTV_HR_ volume in 61 patients with locally advanced cervical cancer was 32 cm^3^, and the 1-year local control rate was 81%. Significant bleeding at the time of applicator removal occurred in four of 214 (1.9%) cases, but all bleeding could be stopped by intravaginal packing [34].

The results of 42 cases of IC/IS using the standard radiotherapy schedule in Japan were reported [9]. The median CTV_HR_ volume at the time of initial brachytherapy was 37 cm^3^, and in all cases, although it was difficult to deliver an adequate dose with conventional ICBT, the 2-year local control rate was 80%. Late gastrointestinal adverse events of grade 3 or higher were observed in three patients (7.1%).

In conclusion, IC/IS has clear advantages in terms of dose distribution compared to ICBT. It is recommended because of its clinical advantages in cases in which the conventional ICBT does not provide sufficient dose to cover the entire CTV_HR_ and cases in which ICBT alone does not provide sufficient dose sparing to the adjacent risk organs. On the other hand, careful attention should be paid to the risk of damage to normal tissues surrounding CTV by interstitial needles.

### QA/QC

A) Quality assurance and control of treatment devices, treatment planning systems, and applicators

For quality control and commissioning of treatment devices and treatment planning systems, refer to the Practical and QA Manual for Sealed Radioactive Source Brachytherapy Based on the Guidelines of the Brachytherapy Committee [3, 35], and for quality assurance required for the initiation of IGBT, refer to the Guidelines for the Introduction of Image-Guided Brachytherapy (IGBT) [7].

B) Applicator and catheter quality control

The novel applicator dedicated to IC/IS has a complex structure with many components, so it should be checked periodically for missing or damaged parts. Since applicators such as disposable plastic interstitial needles cannot be sterilized at their own facility, it is not possible to confirm the position of the radiation source using an actual radiation source beforehand. Therefore, before the start of clinical use, an applicator for QA that is not used for actual patients must be introduced separately to confirm the source anchorage position (referring to the commissioning of applicator reconstruction).

It is advisable to visually confirm that there are no abnormalities such as bends or indentations each time it is used.

## DISCUSSION

Since the introduction of 3D-IGBT, the clinical outcome has dramatically improved compared to the 2D era [19, 36]. However, it was elucidated that even with 3D-IGBT, it is challenging to achieve the recommended dose constraints for CTV_HR_ and OARs, and IC/IS can improve dose distribution, especially for large tumors and/or irregularly shaped tumors [8, 9, 25, 33]. Although the effectiveness of IC/IS has been proposed, it has to be said that the implementation speed of IC/IS is slow, and despite the fact the number of institutions capable of performing IC/IS is gradually increasing, the majority of institutions still cannot perform IC/IS in daily practice, supposedly due to its invasiveness. Although the clinical results of IC/IS have been reported, until now, there have been no guidelines for required equipment, human resources, and procedural guides focusing solely on IC/IS. Therefore, to help spread IC/IS, JASTRO decided to create a practical and comprehensive guide dedicated to IC/IS. Because IC/IS is more invasive than conventional ICBT and deeper sedation is required, when the conditions in each institution allow, it is recommended that radiation oncologists conduct sedation, at least initially, in collaboration with anesthesiologists. This guideline deals with practical patient selection, preparation, procedures, and evidence supporting the effectiveness and safety of IC/IS. The authors hope that this article will help radiation oncologists and medical physicists who wish to start IC/IS and contribute to improving the clinical outcomes of patients suffering from this devastating disease.

Even though the incidence of uterine cervical cancer could be reduced by human papillomavirus vaccination [37], it will be long before most countries are fully vaccinated and it will be one of the major health problems for the next several decades in most countries. In such circumstances, this guideline will help promote IC/IS and increase the curative rate of definitive radiotherapy.

The limitations of this guideline are that most of the information described above is not based on phase III clinical trials but on expert opinions or retrospective studies. However, because of the paucity of phase III clinical trials in the field of brachytherapy, the authors believe that collecting such expert opinions and results from retrospective studies and creating this kind of clinical guideline is valuable.
